# Predictors of Treatment Outcome in an Early Intervention Eating Disorder Sample

**DOI:** 10.1002/eat.24593

**Published:** 2025-11-10

**Authors:** Karina L. Allen, Amelia Austin, Michaela Flynn, Danielle Glennon, Victoria A. Mountford, Amy Brown, Mary Franklin‐Smith, William Rhys Jones, Gabrielle Brady, Nicole Nunes, Frances Connan, Kate Mahony, Lucy Serpell, Ulrike Schmidt

**Affiliations:** ^1^ South London and Maudsley NHS Foundation Trust London UK; ^2^ Centre for Research in Eating and Weight Disorders, Institute of Psychiatry, Psychology and Neuroscience King's College London London UK; ^3^ Mathison Centre for Mental Health Research & Education, Cumming School of Medicine University of Calgary Calgary Canada; ^4^ Inside Out Institute Sydney Australia; ^5^ Sage Clinics Dubai UAE; ^6^ Sussex Partnership NHS Foundation Trust UK; ^7^ Leeds and York Partnership NHS Foundation Trust Leeds UK; ^8^ Central and North West London NHS Foundation Trust London UK; ^9^ North East London NHS Foundation Trust London UK; ^10^ University College London Division of Psychology and Language Sciences London UK

**Keywords:** anorexia nervosa, bulimia nervosa, early intervention, eating disorders, emerging adulthood, other specified feeding or eating disorder, psychological therapy, treatment outcomes

## Abstract

**Objective:**

To examine baseline predictors of treatment completion and clinical outcomes in 16 to 25‐year‐olds referred for early intervention for a recent‐onset eating disorder (ED).

**Method:**

Participants (*n* = 228; 93% female, 63% White British) were drawn from the FREED‐Up study, which evaluated First episode Rapid Early intervention for EDs (FREED) in England across 2017–2018. Measures were completed at baseline and 3‐, 6‐ and 12‐months. Generalized estimating equations were used to identify baseline predictors of treatment completion and symptom remission by 12‐months. Linear mixed models were used to identify baseline predictors of change in global eating disorder examination‐questionnaire (EDE‐Q) scores, binge eating, purging and BMI (anorexia nervosa [AN] only) over 12‐months. Possible predictors included baseline ED symptoms, depression, anxiety, stress, psychosocial impairment, expressed emotion from close others, functioning against personalized goals, age, ethnicity, duration of illness, waiting times and BMI.

**Results:**

There were no significant predictors of treatment completion. Lower stress at baseline predicted increased likelihood of symptom remission by 12 months. Lower purging and psychosocial impairment at baseline predicted lower Global EDE‐Q scores over the following 12 months; lower purging predicted lower rates of binge eating; and lower Global EDE‐Q scores, binge eating and assessment waiting times predicted lower rates of purging. Higher purging at baseline was associated with higher BMI in AN.

**Discussion:**

This study provides new data on predictors of treatment outcomes in an early intervention ED sample. In addition to ED symptoms and waiting times, psychosocial impairment and stress warrant consideration as factors that may influence treatment outcomes.

## 1 Introduction

Outcomes from adult eating disorder (ED) treatments require improvement. Between 20% and 70% of patients discontinue treatment early (Fassino et al. 2009; Linardon et al. 2018) and remission rates are 10%–50% depending on sample, follow‐up and “remission” definition (Kordy et al. 2002; Linardon and Wade 2018; Solmi et al. 2024). There are few consistent predictors of outcome. Less severe ED symptoms are the most robust predictor (Vall and Wade 2015), with psychosocial difficulties, motivation to change, self‐efficacy, age of ED onset and shorter illness duration predicting outcomes less consistently (Solmi et al. 2024; Vall and Wade 2015; Radunz et al. 2020).

Early intervention improves treatment outcomes for 16 to 25‐year‐olds with a recent‐onset ED (Austin et al. 2022; Fukutomi et al. 2020). To date, studies have not examined predictors of outcome within early intervention ED samples. This is important if optimum treatment is to be facilitated for these patients. Early intervention can be defined in different ways but one of the most common definitions is *early detection of emerging disease* followed by *stage‐specific or proportional intervention* (McGorry et al. 2018). The first episode rapid early intervention (FREED) model for EDs uses this definition, and was developed to provide rapid access to stage‐specific, developmentally tailored care for 16 to 25‐year‐olds with an ED of < 3‐years duration (Austin et al. 2022; Fukutomi et al. 2020; Flynn et al. 2021).

The current study aimed to examine predictors of (1) treatment completion and (2) clinical outcomes in 16 to 25‐year‐olds who received FREED early intervention (ED < 3 years). Clinical outcomes included symptom remission (global eating disorder examination [EDE‐Q] score < 2.8, no binge eating or purging over the past 28 days, BMI > 18.5) and changes in global EDE‐Q scores, binge eating and purging episodes, and BMI (AN only) over 12 months. Predictor variables included ED symptoms, impairment, depression/anxiety/stress, psychosocial functioning, age, ethnicity, duration of untreated ED (DUED), waiting times and BMI.

## 2 Method

Data were drawn from the FREED‐Up study (Austin et al. 2022; Fukutomi et al. 2020; Flynn et al. 2021). FREED‐Up participants were 16 to 25‐year‐olds with a *DSM‐5* ED of < 3 years duration. Recruitment occurred through four ED services in England across 2017–2018. All consecutive eligible patients were invited to participate (Flynn et al. 2021).

FREED involves a service model and care package. The service model includes an engagement call to the patient within 48 h of referral and access targets (2 weeks from referral to assessment, 4 weeks from referral to treatment). The care package outlines how standard evidence‐based ED treatment (e.g., CBT‐ED) may be adapted to emerging adults with recent‐onset EDs (Austin et al. 2022; Fukutomi et al. 2020; Flynn et al. 2021). Further information on FREED, FREED‐UP study procedures, and treatment provision is available in (Austin et al. 2022; Flynn et al. 2021).

### 2.1 Participants

Two hundred seventy eight patients were enrolled in FREED‐Up (mean age = 20.19 years [SD 2.39], mean DUED = 17.85 months [SD 10.38]). Given our focus on baseline predictors of outcome, we excluded patients with prior ED treatment (*n* = 50), giving *N* = 228. Most patients with prior treatment were directly transitioning from a child/adolescent ED service (*n* = 17/50.34%) or an adult service in another geographical region (see Supporting Information).

Most participants were female (*n* = 212, 93%). 40% were diagnosed with anorexia nervosa (AN) (*n* = 90), 32% with other specified feeding or eating disorder (OSFED) (*n* = 73), 27% with bulimia nervosa (BN) (*n* = 62), and 1.3% with binge eating disorder (BED) (*n* = 3). Table 1 shows other characteristics, including ethnicity.

**TABLE 1:** Patient characteristics (means [and standard deviations] unless otherwise stated) (*N* = 228).

Age at onset	18.79 (2.46)
Age at assessment	20.19 (2.39)
Ethnicity	
White	63% (*n* = 144)
Black	5% (*n* = 11)
Asian	11% (*n* = 25)
Mixed	7% (*n* = 17)
Other	4% (*n* = 10)
Unknown	9% (*n* = 21)
Duration of untreated eating disorder (DUED) (months)	17.85 (10.38)
Assessment wait (days)	25.07 (26.52)
Treatment wait (days)	56.42 (40.12)
Baseline characteristics	
BMI	20.09 (4.44)
Global EDE‐Q	4.08 (1.21)
Binge eating (% [*n*])	61% (*n* = 140)
Purging (% [*n*])	47% (*n* = 107)
DASS‐depression	11.82 (5.75)
DASS‐anxiety	8.46 (5.37)
DASS‐stress	12.51 (5.09)
CORE‐10	19.66 (7.53)
CIA	32.74 (9.64)
WSAS	20.68 (8.79)
LEE	14.64 (11.17)
PSYCHLOPS	16.00 (3.16)
Treatment completion (% [*n*])	80% (*n* = 182)
Number of treatment sessions	18.64 (12.64)
Full remission by last assessment (% [*n*])	27% (*n* = 61)

Abbreviations: BMI, body mass index; CIA, clinical impairment assessment; CORE‐10, clinical outcomes in routine evaluation‐10; DASS, depression anxiety stress scale; EDE‐Q, eating disorder examination‐questionnaire; LEE, levels of expressed emotion scale; PSCHLOPS, psychological outcome profiles; WSAS, work and social adjustment scale.

### 2.2 Procedures

At baseline, participants provided written informed consent and completed a semi‐structured interview to confirm illness onset/DUED. They also completed self‐report questionnaires. Participants were invited to complete follow‐up questionnaires after 3, 6 and 12 months (regardless of treatment status). To allow for consistency in BMI data, self‐reported weight was used to calculate BMI at baseline and follow‐ups.

### 2.3 Measures

#### 2.3.1 Patient‐Report Questionnaires

Participants completed the eating disorder examination‐questionnaire (EDE‐Q) (Fairburn and Beglin 2008; Mond et al. 2008), depression, anxiety and stress scale–21 item (DASS‐21) (Lovibond and Lovibond 1995), clinical outcomes in routine evaluation‐10 item (CORE‐10) (Barkham et al. 2013), clinical impairment assessment (CIA) (Bohn et al. 2008), work and social adjustment scale (WSAS) (Mundt et al. 2002), levels of expressed emotion scale (LEE) (Cole and Kazarian 1988), and psychological outcome profiles (PSYCHLOPS) (Ashworth et al. 2004). These have well‐established psychometric properties. Further details are provided in Supporting Information. Participants also self‐reported demographic information, including ethnicity, and weight. Due to the small number of participants in each non‐white ethnic group (Table 1), ethnicity was considered a categorical variable (white vs. non‐white) with acknowledgment of the limitations of this approach.

The EDE‐Q was used as a predictor and outcome variable, as was BMI for patients with AN. Other self‐report variables were predictors only.

#### 2.3.2 Clinician/Researcher‐Reported Variables

Age at assessment, age at onset, assessment wait (referral to assessment), treatment wait (referral to first treatment session) and DUED (ED onset to assessment) were recorded by a FREED‐Up researcher. Clinicians measured baseline height (for BMI) and reported ED diagnosis, number of treatment sessions, and whether treatment was completed. “Treatment completion” meant that the clinician and patient mutually agreed to end treatment and all treatment stages had been completed.

### 2.4 Analyses

Analyses were conducted in SPSS 29.

Data were not missing completely at random (Little's MCAR *p* < 0.001). Whilst < 1% of variables were missing data at baseline, 22%–38% of variables were missing data over follow‐up. 20% of participants (*n* = 44) did not complete any post‐baseline assessments. Of these, 73% (*n* = 32) were individuals who didn't complete treatment while 27% (*n* = 12) completed treatment but not assessments. The only significant predictor of missingness was treatment completion, so this was included in models as a covariate (excepting models predicting treatment completion). This is consistent with prior FREED‐Up analyses (Austin et al. 2022).

Generalized estimating equations (GEE) were used to examine predictors of treatment completion and remission by 12 months (Y/N). The remission variable used last observation carried forward for missing data. Thus, participants with no data post‐baseline (20%) were classified as non‐remitted.

Linear mixed models (LMM) were used to examine predictors of Global EDE‐Q scores and BMI (AN; *n* = 90) from baseline to 12‐month follow‐up. Generalized LMMs with negative binomial regression were used to examine predictors of binge eating and purging. Negative binomial regression can account for the non‐normal distribution of binge/purge episodes (i.e., a skew towards zero).

GEEs and LMMs make use of all available data and can account for repeated measurements and the clustering of patients within services (Stroup et al. 2024). All models included random effects of service and entered time as a predictor (four categories: baseline, 3‐months, 6‐months, 12‐months). Further detail is in Supporting Information.

Initially, univariate associations were considered between each predictor and outcome. For LMM, these indicated if the baseline variable predicted overall/average levels of the outcome over the subsequent 12 months. Predictor × time LMM interactions were then specified to test if the baseline variable predicted degree/rate of *change* in the outcome over 12 months.

Univariate predictors reaching statistical significance were entered into multivariate models. In total, 87 univariate tests were conducted and a Bonferroni‐adjusted *p* value was applied: adjusted *p* < 0.005.

## 3 Results

### 3.1 Descriptive Statistics

Descriptive characteristics are summarized in Table 1. Details for AN and BN/BED/OSFED patients separately are provided in Table S1.

### 3.2 Predictors of Treatment Completion and Full Symptom Remission

#### 3.2.1 Treatment Completion

80% of patients completed treatment. There were no significant predictors of completion (Table S2).

#### 3.2.2 Symptom Remission

27% of participants remitted by 12 months. Baseline BMI, global EDE‐Q, DASS‐depression, DASS‐stress, CORE‐10, CIA, WSAS and PSYCHLOPS were significant predictors in univariate GEE (Table S2). In the multivariate GEE, only DASS‐stress entered the model at *p* < 0.005, *B =* −0.37 (95% CI: −0.61, −0.13) (Table S3).

### 3.3 Predictors of ED Symptom Change

#### 3.3.1 Global EDE‐Q Scores

There was a significant reduction over time in global EDE‐Q scores (*p* < 0.001). Baseline BMI, binge eating, purging, DASS‐depression, DASS‐anxiety, DASS‐stress, CORE‐10, CIA, WSAS and PSYCHLOPS were significant univariate predictors of Global EDE‐Q scores over 12 months (Table S4). No baseline predictors interacted significantly with time. In the multivariate LMM, vomiting, CIA and PSYCHLOPS remained significant (Table 2). Higher baseline levels predicted higher Global EDE‐Q scores.

**TABLE 2:** Baseline predictors of changes in global EDE‐Q scores, binge eating, purging and BMI over the 12‐month study period—multivariate associations controlling for treatment completion.

	Estimate	*p*	95% CI	*p* interaction^a^
Global EDE‐Q scores (*n* = 228)				
Time (12‐months vs. baseline)	−1.55	< 0.001*	−1.93, −1.18	
Treatment completion (Y vs. N)	0.10	0.303	−0.11, 0.36	
BMI	0.02	0.202	−0.01, 0.04	
Binge eating/month	0.10	0.186	−0.01, 0.02	
Purging/month	0.02	0.004*	0.01, 0.02	
DASS‐depression	−0.02	0.170	−0.06, 0.01	
DASS‐anxiety	0.01	0.706	−0.02, 0.04	
DASS‐stress	0.03	0.06	−0.01, 0.07	
CORE‐10	0.01	0.336	−0.01, 0.04	
CIA	0.04	< 0.001*	0.02, 0.06	
WSAS	−0.01	0.688	−0.02, 0.01	
PSYCHLOPS	0.10	< 0.001*	0.05, 0.15	
Binge eating/month (*n* = 228)				
Time (12‐months vs. baseline)	−1.39	< 0.001*	−1.65, −1.13	
Treatment completion (Y vs. N)	−0.15	0.408	−0.50., 0.20	
BMI	0.05	0.008	0.01, 0.09	—
Purging/month	0.03	< 0.001*	0.02, 0.05	< 0.001*
CIA	0.01	0.949	−0.02, 0.03	—
LEE	0.02	0.048	0.01, 0.03	—
WSAS	0.02	0.107	−0.01, 0.05	—
Purging/month (*n* = 228)				
Time (12‐months vs. baseline)	−1.73	< 0.001*	−2.54, −0.92	
Treatment completion (Y vs. N)	−0.83	< 0.001*	−1.23, 0.42	
Age at assessment	−0.29	0.009	−0.51, −0.07	0.222
Age at onset	0.19	0.039	0.01, 0.37	0.023
Assessment wait (days)	0.04	< 0.001*	0.02, 0.06	—
Treatment wait (days)	0.01	0.101	−0.01, 0.02	—
Global EDE‐Q score	0.35	< 0.001*	0.17, 0.53	0.007
Binge eating/month	0.10	< 0.001*	0.05, 0.16	
BMI (including AN patients only; *n* = 90)				
Time (12‐months vs. baseline)	2.30	< 0.001*	1.14, 3.46	
Treatment completion (Y vs. N)	−0.47	0.208	−1.07, 0.13	
Purging/month	0.04	0.002*	0.02, 0.07	

Abbreviations: BMI, body mass index; CIA, clinical impairment assessment; CORE‐10, clinical outcomes in routine evaluation‐10; DASS, depression anxiety stress scale; EDE‐Q, eating disorder examination‐questionnaire; LEE, levels of expressed emotion scale; PSCHLOPS, psychological outcome profiles; WSAS, work and social adjustment scale.

^a^ Interaction terms were only entered if significant at *p* < 0.005 in univariate models.

* *p* < 0.005.

#### 3.3.2 Binge Eating

Binge eating reduced significantly over time (*p < 0*.001). Baseline BMI, purging, CIA, WSAS and LEE scores were significant univariate predictors of binge eating over 12 months (Table S4). There was a significant univariate interaction between baseline purging and time (*p* < 0.001). In the multivariate LMM, purging was the only significant predictor. The purging × time interaction also remained statistically significant (Table 2).

To interpret this interaction, baseline purging was converted to a categorical variable. Details are included in Supporting Information and Figure S1. The group with the highest baseline purging reported higher binge eating than other groups, and did not report reductions in binge eating until after 6 months. Participants with no/minimal baseline purging reported lower rates of binge eating overall, and reductions in binges from baseline onwards.

#### 3.3.3 Purging

Purging reduced significantly over time (*p* < 0.001). Age at onset and assessment, assessment and treatment waiting times, baseline global EDE‐Q scores and baseline binge eating significantly predicted purging over the subsequent 12 months. There were significant interactions with time for age at onset and assessment, and Global EDE‐Q scores (Table S4).

In the multivariate model, assessment waiting time, baseline global EDE‐Q scores, and baseline binge eating were significant predictors (Table 2). Longer assessment waits, higher global EDE‐Q scores and greater binge eating predicted higher rates of purging over 12 months. There were no significant interactions with time.

#### 3.3.4 BMI

For patients with AN, BMI increased significantly over time (*p* < 0.001). Only baseline purging predicted BMI over the subsequent 12 months, without a significant interaction with time (Table 2, Table S4). Higher purging at baseline was associated with higher BMI.

## 4 Discussion

This study examined predictors of treatment completion and outcomes in an early intervention ED sample. Findings partially converge with those from non‐early intervention groups. For example, as reported previously (Vall and Wade 2015), less severe ED symptoms at baseline predicted improved clinical outcomes. Higher baseline purging predicted higher global EDE‐Q scores, on average over 12 months, and higher baseline global EDE‐Q and binge scores predicted higher rates of purging. Higher baseline purging predicted higher rates of binge eating, but also different rates of change in binge eating, over the 12 months.

Previous studies have also found links between baseline psychosocial distress or interpersonal difficulties and subsequent outcomes (Vall and Wade 2015). In this early intervention sample, higher impairment related to an ED (CIA and PSYCHLOPS scores) predicted higher global EDE‐Q scores over the subsequent 12 months. Higher stress at baseline also predicted a lower likelihood of symptom remission by 12 months. Remission rates were 23% for AN and 29% for non‐AN patients, which are similar to, or slightly higher than, equivalent figures for non‐early intervention samples (e.g., 16%–27%; Mountford et al. 2021). Stress can be conceptualized in multiple ways and our analyses used the DASS‐stress scale. This assesses difficulty relaxing and being easily upset/agitated, irritable/over‐reactive and impatient over the past week (Lovibond and Lovibond 1995). Some of these experiences may correlate with dietary restraint and different results may emerge if using different measures of stress (e.g., exposure to stressors). Nevertheless, the findings speak to psychosocial stress/distress being important in the pattern of change for early intervention ED patients and warranting attention by clinicians.

We previously identified links between longer DUED and increased likelihood of binge eating within FREED‐Up (Austin et al. 2025). Here, DUED did not significantly predict changes in ED symptoms over treatment. This suggests that patients with recent‐onset EDs can benefit from early intervention regardless of precise illness length. However, we did find a small positive association between longer waiting time for assessment and higher rates of purging over the following 12 months. This warrants further attention, particularly as FREED significantly reduced waiting times at participating services (Flynn et al. 2021). It is unclear if this finding would generalize to samples with longer waiting times, and/or if consistently meeting FREED access targets (2 weeks from referral to assessment) would further improve outcomes.

There were no significant predictors of treatment completion. 80% of participants completed treatment, a higher proportion than seen in most ED samples (Fassino et al. 2009; Linardon et al. 2018). Clinicians working with early intervention ED patients should be encouraged to focus on high rates of treatment completion regardless of patient characteristics.

Identified predictors had small effect sizes, which is consistent with prior work with non‐early intervention ED samples (Vall and Wade 2015). It is increasingly recognized that psychosocial factors may only partially explain psychiatric outcomes. Genetic, metabolic and biomarker data are important considerations for future studies (García‐Gutiérrez et al. 2020). Patterns of symptom change, including early response to treatment, may also predict outcomes more fully than baseline symptoms alone (e.g., McClure et al. 2025).

Other limitations include a lack of power to consider differences between diagnostic subgroups and not having information on specific OSFED diagnoses. We use a particular conceptualization of early intervention, and arguably 3 years of illness is not “early intervention” for many young people with EDs. Predictors of outcome may be different if different early intervention models are used.

In conclusion, we found that pre‐treatment binge eating, purging, ED psychopathology (global EDE‐Q), stress (DASS‐stress), impairment (CIA/WSAS), and waiting times predicted treatment outcomes for 16 to 25‐year‐olds receiving early intervention for an ED. Clinicians can be encouraged by high rates of treatment completion for this patient group. Further research is needed to consider interactive models that incorporate a wider range of data sources, and the optimum time period for early intervention (in terms of DUED) and rapid access (in terms of waiting times after referral).

## Author Contributions

**Karina L. Allen:** writing – original draft, formal analysis, methodology, investigation, funding acquisition, conceptualization. **Amelia Austin:** writing – review and editing, project administration, methodology, investigation, conceptualization. **Michaela Flynn:** writing – review and editing, validation, project administration, methodology, investigation. **Danielle Glennon:** writing – review and editing, methodology, investigation. **Victoria A. Mountford:** writing – review and editing, methodology, investigation. **Amy Brown:** writing – review and editing, methodology, investigation. **Mary Franklin‐Smith:** writing – review and editing, investigation. **William Rhys Jones:** writing – review and editing, investigation. **Gabrielle Brady:** writing – review and editing, investigation. **Nicole Nunes:** writing – review and editing, investigation. **Frances Connan:** writing – review and editing, investigation. **Kate Mahony:** writing – review and editing, investigation. **Lucy Serpell:** writing – review and editing, investigation. **Ulrike Schmidt:** writing – review and editing, supervision, methodology, funding acquisition, conceptualization.

## Conflicts of Interest

The authors declare no conflicts of interest.

## Supporting information


**Figure S1:** Mean binge eating episodes over time, by purging frequency at baseline. Purging frequency/month was 0 for the no baseline purging group, 1–3 for the low group, 4–16 for the moderate group and > 16 for the high group.

**Data S1:** Supporting Information.

## Data Availability

The data that support the findings of this study are available from the corresponding author upon reasonable request.
