# Supplementary figures and images for: Predictors of Treatment Outcome in an Early Intervention Eating Disorder Sample
Source: Int J Eat Disord. 2025 Nov 10;59(3):574–80. doi: 10.1002/eat.24593 (PMC12979957; doi:10.1002/eat.24593)

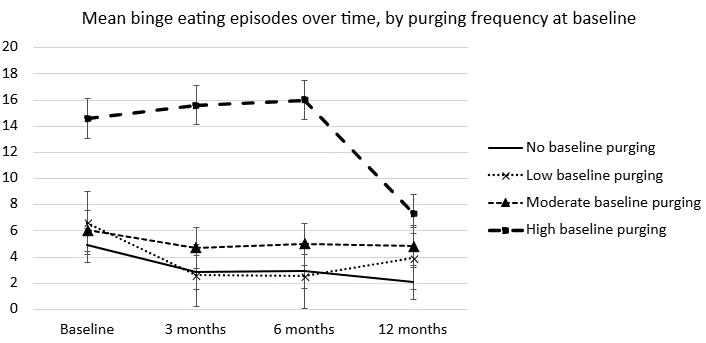

Supplement: Supplementary file 1 — Figure S1: Mean binge eating episodes over time, by purging frequency at baseline. Purging frequency/month was 0 for the no baseline purging group, 1–3 for the low group, 4–16 for the moderate group and > 16 for the high group. [file EAT-59-574-s001.gif]
