# Supplementary material for: Predictors of Treatment Outcome in an Early Intervention Eating Disorder Sample
Source: Int J Eat Disord. 2025 Nov 10;59(3):574–80. doi: 10.1002/eat.24593 (PMC12979957; doi:10.1002/eat.24593)
Supplement: Supplementary file 2 — Data S1: Supporting Information. [file EAT-59-574-s002.docx]

**Supplementary information**

**Participants with prior eating disorder treatment**

Most patients with prior ED treatment (n=50) were directly transitioning from a child/adolescent ED service (n=17/50,34%) or an adult service in another geographical region. This precluded consideration of baseline predictors of treatment outcome. Consistent with initial treatment effects, these 50 patients had lower mean scores on all clinical variables at the initial FREED-Up assessment than patients entering treatment for the first time. Patients with prior treatment also had a significantly younger age of ED onset (M=17 years [SD 1.8] vs. 19 years [SD 2.4]) than patients entering treatment for the first time, with a significantly longer duration of illness (26 months [SD 9.7] vs. 17 months [SD 9.7]) [8,9].

**Questionnaire measures**

**Eating Disorder Examination-Questionnaire (EDE-Q) [12].** This assesses ED cognitions and behaviours over the past 28 days. The Global score (range=0-6) is an overall index of ED psychopathology; scores ≥2.8 suggest clinically concerning symptoms [13]. Behavioural items determine presence and frequency of objective binge eating and purging (self-induced vomiting + laxative misuse).

**Depression, Anxiety and Stress Scale–21 item (DASS-21) [14].** This assesses symptoms of depression, anxiety and stress over the past 7 days. Depression, anxiety and stress scores were considered as separate variables (range=0-42). The Depression scale assesses feelings of dysphoria, hopelessness, lack of interest/involvement, anhedonia, self-criticism, and inertia. The Anxiety scale assesses autonomic arousal, muscle tension, situational anxiety, and subjective anxiety. The Stress scale assesses difficulty relaxing, nervous arousal, being easily upset/agitated, being irritable/over-reactive and impatience [14].

**Clinical Outcomes in Routine Evaluation-10 item (CORE-10) [15].** This assesses anxiety and depressive symptoms. Total scores range from 0-3. Scores ≥1 are clinically significant.

**Clinical Impairment Assessment (CIA) [16].** This is a 16-item measure of psychosocial impairment related to an ED, with total scores between 0-48.

**Work and Social Adjustment Scale (WSAS) [17].** This is a 5-item measure of functional impairment due to illness (an ED). Total scores range from 0-40.

**Levels of Expressed Emotion Scale (LEE) [18].** This measures perceived expressed emotion from a close other. It includes 60 true/false items across four subscales: attitude towards illness, emotional response, intrusiveness, and tolerance/expectations. We used a total score averaged across subscales (range=0-60).

**The Psychological Outcome Profiles (PSYCHLOPS) [19].** This is an individualised outcome measure that evaluates function against patient-generated outcomes. Examples in FREED-Up include “commit to my studies” and “get a good night's sleep” [8]. Total scores range from 0-20.

**Analyses**

Generalised estimating equations (GEEs) were used to examine predictors of treatment completion and remission (categorical outcomes: Y/N). Linear mixed models (LMMs) were used to examine predictors of Global EDE-Q scores and BMI (AN patients only; n=90) from baseline to 12-month follow-up; and generalised LMMs with negative binomial regression were used to examine predictors of binge eating and purging from baseline to 12-month follow-up. Negative binomial regression can account for the non-normal distribution of binge/purge episodes (i.e., with a skew towards zero).

As noted in the main manuscript, GEEs, LMMs and generalised LMMs make use of all available data and can account for repeated measurements and the clustering of patients within services. All models included random effects of service. Fixed effects included the predictor variables of interest and time (4 categories: baseline, 3-months, 6-months, 12-months).

Random effects take into account unmeasured sources of variance and recognise that samples are drawn from a larger population. In this study, the four FREED-Up services provide a source of potential cross-site variance; there may be site-specific factors which influence associations between predictor and outcome variables. Moreover, these four ED services are a sub-set of the larger number of ED services in the UK. By adding a random effect of service, LMMs take into account the clustering of patients within services and any service-level effects on outcomes. Random effects also allow models to recognise that included participants/services are a subset of a wider population, which is not measured but which results may also apply to. Finally, with longitudinal data, random effects help account for the expected correlation of data over time.

Fixed effects test associations between predictor variables of interest and outcomes. Categorical fixed effects compare outcomes across mutually exclusive groups, for example, treatment completers compared to non-completers. Continuous fixed effects capture associations between the predictor (e.g., depression scores) and outcome (e.g., binge eating episodes) variables.

Predictor x time interaction effects consider how the effects of a predictor variable may influence changes in the outcome over time. In the example below, the purging x time interaction shows that different levels of baseline purging have a different effect on changes in binge eating over time. Further information on LMMs can be found in [24].

**Purging x time interaction in the prediction of binge eating.** To interpret the purging x time interaction in the multivariate model for binge eating, baseline purging was converted to a categorical variable. We applied an adjusted quartile split due to 45% of the sample reporting no baseline purging: no purging (n=201, 45%), minimal (1-3 episodes/month; n=18, 8%), moderate (4-16 episodes/month; n=55, 24%) and high (>16 episodes/month; n=53, 23%). This categorical variable also interacted significantly with time to predict changes in binge eating (*p*<.001). As shown in Supplementary Figure 1, participants with high purging at baseline reported higher levels of binge eating across the study period and experienced, on average, a slight increase in binge eating from baseline to 6-months before a decrease by 12-months. Participants with no/minimal purging experienced reductions in binges over time, with the exception of a slight increase in binges between 6 and 12-months for the group with minimal baseline purging. Participants with moderate baseline purging reported relatively stable binge eating over time.

*Supplementary Figure 1.*

Mean binge eating episodes over time, by purging frequency at baseline. Purging frequency/month was 0 for the no baseline purging group, 1-3 for the low group, 4-16 for the moderate group and >16 for the high group.

Supplementary Table 1

*Patient characteristics for participants with anorexia nervosa (AN) compared to bulimia nervosa (BN), binge eating disorder (BED) or an other specified feeding or eating disorder (OSFED) (means [and standard deviations] unless otherwise stated)*

|  | AN (n=90) | BN, BED and OSFED (n=138) |
| --- | --- | --- |
| Age at onset | 18.51 (2.37)_a_ | 18.99 (2.52)_b_ |
| Age at assessment | 19.93 (2.22)_a_ | 20.38 (2.49)_b_ |
| Ethnicity |  |  |
| White | 67% (n=60) | 61% (n=84) |
| Black | 3% (n=3) | 6% (n=8) |
| Asian | 11% (n=10) | 11% (n=15) |
| Mixed | 6% (n=5) | 9% (n=12) |
| Other | 4% (n=4) | 4% (n=6) |
| Unknown | 9% (n=8) | 9% (n=13) |
| Duration of Untreated Eating Disorder (DUED) (months) | 17.50 (10.62) | 18.11 (10.22) |
| Assessment wait (days) | 22.76 (18.53)_a_ | 26.75 (31.01)_b_ |
| Treatment wait (days) | 51.87 (33.46) | 59.77 (44.19) |
| Baseline characteristics: |  |  |
| BMI | 16.62 (1.27)_a_ | 22.63 (4.19)_b_ |
| Global EDE-Q | 3.73 (1.41)_a_ | 4.33 (0.98)_b_ |
| Binge eating (% [n]) | 40% (n=36)_a_ | 75% (n=104)_b_ |
| Purging (% [n]) | 16% (n=14)_a_ | 67% (n=93)_b_ |
| DASS-Depression | 11.52 (5.54) | 12.04 (5.90) |
| DASS-Anxiety | 8.69 (5.24) | 8.29 (5.47) |
| DASS-Stress | 12.77 (5.07) | 12.32 (5.11) |
| CORE-10 | 19.34 (7.27) | 19.90 (7.73) |
| CIA | 32.19 (10.31) | 33.13 (9.14) |
| WSAS | 21.31 (8.00) | 20.23 (9.32) |
| LEE | 14.27 (10.88) | 15.30 (11.30) |
| PSYCHLOPS | 15.96 (3.26) | 16.04 (3.10) |
| Treatment completion (% [n]) | 80% (n=72) | 80% (n=110) |
| Number of treatment sessions | 21.99 (15.90)_a_ | 14.71 (10.18)_b_ |
| Full remission by last assessment (% [n]) | 23% (n=21) | 29% (n=40) |

BMI = Body Mass Index; CIA = Clinical Impairment Assessment; CORE-10 = Clinical Outcomes in Routine Evaluation-10; DASS = Depression Anxiety Stress Scale; EDE-Q = Eating Disorder Examination-Questionnaire; LEE = Levels of Expressed Emotion Scale; PSCHLOPS = Psychological Outcome Profiles; WSAS = Work and Social Adjustment Scale

Different subscripts denote statistically significant group differences (one-way ANOVA, *p*<.05)

Supplementary Table 2

*Univariate associations between baseline predictor variables and treatment completion and full symptom remission (y/n)*

|  | **B** | ***P*** | **95% CI** |
| --- | --- | --- | --- |
| **Treatment completion** | | | |
| Age at assessment | 0.09 | .146 | -0.03, 0.20 |
| Age at onset | 0.10 | .112 | -0.02, 0.22 |
| Ethnicity (white vs. non-white) | 0.21 | .549 | -0.91, 0.48 |
| Assessment wait | -0.01 | .602 | -0.01, 0.01 |
| Treatment wait | -0.01 | .198 | -0.01, 0.01 |
| DUED | -0.01 | .819 | -0.03, 0.03 |
| BMI | 0.01 | .888 | -0.06, 0.06 |
| Global EDE-Q score | 0.01 | .932 | -0.24, 0.26 |
| Binge eating/month | 0.02 | .305 | -0.02, 0.05 |
| Purging/month | -0.01 | .393 | -0.04, 0.01 |
| DASS-depression | -0.01 | .701 | -0.07, 0.05 |
| DASS-anxiety | -0.06 | .075 | -0.13, 0.01 |
| DASS-stress | 0.01 | .859 | -0.06, 0.08 |
| CORE-10 | -0.02 | .368 | -0.07, 0.03 |
| CIA | 0.01 | .899 | -0.04, 0.04 |
| WSAS | -0.02 | .468 | -0.06, 0.03 |
| LEE | -0.02 | .171 | -0.04, 0.01 |
| PSYCHLOPS | 0.02 | .733 | -0.09, 0.13 |
| **Full symptom remission** |  |  |  |
| Age at assessment | -0.19 | .027 | -0.36, -0.02 |
| Age at onset | -0.16 | .063 | -0.32, 0.01 |
| Ethnicity (white vs. non-white) | 0.50 | .328 | -0.50, 1.51 |
| Assessment wait | -0.01 | .860 | -0.01, 0.01 |
| Treatment wait | 0.01 | .191 | -0.01, 0.01 |
| DUED | 0.01 | .947 | -0.04, 0.04 |
| BMI | -0.24 | <.001* | -0.37, -0.10 |
| Global EDE-Q score | -3.15 | <.001* | -4.92, -1.38 |
| Binge eating/month | -0.14 | .041 | -0.28, -0.01 |
| Purging/month | -0.08 | .024 | -0.16, -0.01 |
| DASS-depression | -0.18 | <.001* | -0.27, -0.09 |
| DASS-anxiety | -0.16 | .008 | -0.27, -0.04 |
| DASS-stress | -0.19 | <.001* | -0.28, -0.09 |
| CORE-10 | -0.14 | <.001* | -0.21, -0.06 |
| CIA | -0.20 | <.001* | -0.27, -0.13 |
| WSAS | -0.12 | .001* | -0.20, -0.05 |
| LEE | -0.03 | .172 | -0.07, 0.01 |
| PSYCHLOPS | -0.28 | <.001* | -0.43, -0.12 |

BMI = Body Mass Index; CIA = Clinical Impairment Assessment; CORE-10 = Clinical Outcomes in Routine Evaluation-10; DASS = Depression Anxiety Stress Scale; EDE-Q = Eating Disorder Examination-Questionnaire; LEE = Levels of Expressed Emotion Scale; PSCHLOPS = Psychological Outcome Profiles; WSAS = Work and Social Adjustment Scale **p*<.005

Supplementary Table 3

*Multivariate associations between baseline predictor variables and full symptom remission (y/n)*

| **Full symptom remission** | **B** | ***P*** | **95% CI** |
| --- | --- | --- | --- |
| BMI | 0.01 | .953 | -0.25, 0.26 |
| Global EDE-Q score | -2.27 | .012 | -4.05, -0.49 |
| DASS-depression | -0.10 | .394 | -0.32, 0.13 |
| DASS-stress | -0.37 | .003* | -0.61, -0.13 |
| CORE-10 | 0.14 | .200 | -0.08, 0.37 |
| CIA | -0.12 | .075 | -0.25, 0.01 |
| WSAS | 0.01 | .994 | -0.15, 0.15 |
| PSYCHLOPS | 0.22 | .007 | 0.06, 0.39 |

BMI = Body Mass Index; CIA = Clinical Impairment Assessment; CORE-10 = Clinical Outcomes in Routine Evaluation-10; DASS = Depression Anxiety Stress Scale; EDE-Q = Eating Disorder Examination-Questionnaire; PSCHLOPS = Psychological Outcome Profiles; WSAS = Work and Social Adjustment Scale

**p*<.005

Supplementary Table 4

*Baseline predictors of changes in Global EDE-Q scores, binge eating, purging and BMI over the 12-month study period – Univariate associations controlling for treatment completion*

|  | **Estimate** | ***P*** | **95% CI** | ***P* interaction** |
| --- | --- | --- | --- | --- |
| **Global EDE-Q scores (n=228)** |  |  |  |  |
| Age at assessment | 0.04 | .087 | -0.01, 0.08 | .100 |
| Age at onset | 0.01 | .727 | -0.04, 0.05 | .172 |
| Ethnicity (white vs. non-white) | 0.13 | .249 | -0.01, 0.35 | .956 |
| Assessment wait | 0.01 | .255 | -0.01, 0.01 | .710 |
| Treatment wait | 0.01 | .404 | -0.01, 0.01 | .637 |
| DUED | 0.01 | .037 | .01, 0.02 | .423 |
| BMI | 0.04 | .003* | 0.01, 0.06 | .492 |
| Binge eating/month | 0.04 | <.001* | 0.02, 0.05 | .563 |
| Purging/month | 0.02 | <.001* | 0.01, 0.03 | .590 |
| DASS-depression | 0.08 | <.001* | 0.06, 0.10 | .731 |
| DASS-anxiety | 0.08 | <.001* | 0.06, 0.11 | .728 |
| DASS-stress | 0.10 | <.001* | 0.08, 0.12 | .876 |
| CORE-10 | 0.07 | <.001* | 0.05, 0.08 | .334 |
| CIA | 0.07 | <.001* | 0.06, 0.08 | .506 |
| WSAS | 0.06 | <.001* | 0.04, 0.07 | .606 |
| LEE | 0.02 | .046 | 0.01, 0.03 | .890 |
| PSYCHLOPS | 0.19 | <.001* | 0.16, 0.23 | .824 |
| **Binge eating/month (n=228)** | | | | |
| Age at assessment | -0.04 | .978 | -0.01, 0.08 | .294 |
| Age at onset | -0.01 | .077 | -0.03, 0.05 | .190 |
| Ethnicity (white vs. non-white) | -0.17 | .107 | -0.04, 0.37 | .955 |
| Assessment wait | 0.21 | .057 | -0.01, 0.42 | .990 |
| Treatment wait | 0.01 | .505 | -0.01, 0.03 | .986 |
| DUED | 0.01 | .027 | 0.01, 0.02 | .097 |
| BMI | 0.07 | <.001* | 0.05, 0.10 | .006 |
| Global EDE-Q score | 0.18 | .008 | 0.05, 0.32 | .704 |
| Purging/month | 0.03 | <.001* | 0.02, 0.04 | <.001* |
| DASS-depression | 0.02 | .016 | 0.01, 0.04 | .383 |
| DASS-anxiety | -0.01 | .711 | -0.02, 0.02 | .494 |
| DASS-stress | 0.01 | .823 | -0.02, 0.02 | .269 |
| CORE-10 | 0.01 | .080 | -0.01, 0.02 | .273 |
| CIA | 0.02 | <.001* | 0.01, 0.03 | .963 |
| WSAS | 0.02 | <.001* | 0.01, 0.03 | .673 |
| LEE | 0.02 | .002* | 0.01, 0.04 | .672 |
| PSYCHLOPS | 0.03 | .043 | 0.01, 0.06 | .935 |
| **Purging/month (n=228)** | | | | |
| Age at assessment | -0.11 | .<.001* | -0.15, -0.07 | .002* |
| Age at onset | -0.08 | <.001* | -0.12, -0.03 | .002* |
| Ethnicity (white vs. non-white) | -0.06 | .558 | -0.26, 0.14 | .900 |
| Assessment wait | 0.01 | <.001* | -0.01, -0.01 | .789 |
| Treatment wait | 0.01 | <.001* | -0.01, -0.01 | .701 |
| DUED | -0.01 | .020 | -0.02, -0.01 | .031 |
| BMI | 0.01 | .129 | -0.55, 0.06 | .<.001* |
| Global EDE-Q score | 035 | <.001* | 0.18, 0.51 | <.001* |
| Binge eating/month | 0.08 | <.001* | 0.07, 0.10 | .032 |
| DASS-depression | 0.02 | .015 | 0.01, 0.04 | .149 |
| DASS-anxiety | 0.01 | .660 | -0.02, 0.02 | .843 |
| DASS-stress | -0.03 | .012 | -0.04, -0.01 | .702 |
| CORE-10 | 0.01 | .036 | 0.01, 0.03 | .883 |
| CIA | 0.01 | .895 | -0.01, 0.02 | .854 |
| WSAS | 0.01 | .758 | -0.01, 0.01 | .412 |
| LEE | 0.01 | .040 | 0.01, 0.03 | .650 |
| PSYCHLOPS | 0.02 | .260 | -0.01, 0.05 | .177 |
| **BMI (including AN patients; n=90)** | | | | |
| Age at assessment | 0.02 | .685 | -0.07, 0.10 | .897 |
| Age at onset | -0.03 | .422 | -0.12, 0.05 | .944 |
| Ethnicity (white vs. non-white) | -0.01 | .995 | -0.40, 0.40 | .301 |
| Assessment wait | 0.01 | .277 | -0.01, 0.02 | .362 |
| Treatment wait | 0.01 | .293 | -0.01, 0.01 | .864 |
| DUED | -0.01 | .542 | -0.03, 0.01 | .562 |
| Global EDE-Q score | 0.13 | .054 | -0.01, 0.26 | .583 |
| Binge eating/month | 0.02 | .267 | -0.02, 0.07 | .475 |
| Purging/month | 0.04 | .002* | 0.02, 0.07 | .096 |
| DASS-depression | 0.04 | .195 | -0.02, 0.10 | .289 |
| DASS-anxiety | 0.04 | .155 | -0.02, 0.10 | .882 |
| DASS-stress | 0.01 | .923 | -0.06, 0.07 | .141 |
| CORE-10 | 0.03 | .148 | -0.01, 0.08 | .493 |
| CIA | 0.01 | .558 | -0.02, 0.04 | .692 |
| WSAS | -0.02 | .253 | -0.06, 0.02 | .778 |
| LEE | 0.02 | .169 | -0.01, 0.04 | .524 |
| PSYCHLOPS | 0.07 | .176 | -0.03, 0.18 | .295 |

**p*<.005
